# Slow Physical Growth, Delayed Reflex Ontogeny, and Permanent Behavioral as Well as Cognitive Impairments in Rats Following Intra-generational Protein Malnutrition

**DOI:** 10.3389/fnins.2015.00446

**Published:** 2015-12-08

**Authors:** Aijaz A. Naik, Ishan K. Patro, Nisha Patro

**Affiliations:** ^1^School of Studies in Neuroscience, Jiwaji UniversityGwalior, India; ^2^School of Studies in Zoology, Jiwaji UniversityGwalior, India

**Keywords:** intra-generational protein malnutrition, cliff avoidance, Morris water maze, elevated plus maze, open field test, anxiety

## Abstract

Environmental stressors including protein malnutrition (PMN) during pre-, neo- and post-natal age have been documented to affect cognitive development and cause increased susceptibility to neuropsychiatric disorders. Most studies have addressed either of the three windows and that does not emulate the clinical conditions of intra-uterine growth restriction (IUGR). Such data fail to provide a complete picture of the behavioral alterations in the F1 generation. The present study thus addresses the larger window from gestation to F1 generation, a new model of intra-generational PMN. Naive Sprague Dawley (SD) dams pre-gestationally switched to LP (8% protein) or HP (20% protein) diets for 45 days were bred and maintained throughout gestation on same diets. Pups born (HP/LP dams) were maintained on the respective diets post-weaningly. The present study aimed to show the sex specific differences in the neurobehavioral evolution and behavioral phenotype of the HP/LP F1 generation pups. A battery of neurodevelopmental reflex tests, behavioral (Open field and forelimb gripstrength test), and cognitive [Elevated plus maze (EPM) and Morris water maze (MWM)] assays were performed. A decelerated growth curve with significantly restricted body and brain weight, delays in apparition of neuro-reflexes and poor performance in the LP group rats was recorded. Intra-generational PMN induced poor habituation-with-time in novel environment exploration, low anxiety and hyperactive like profile in open field test in young and adult rats. The study revealed poor forelimb neuromuscular strength in LP F1 pups till adulthood. Group occupancy plots in MWM test revealed hyperactivity with poor learning, impaired memory retention and integration, thus modeling the signs of early onset Alzehemier phenotype. In addition, a gender specific effect of LP diet with severity in males and favoring female sex was also noticed.

## Introduction

Developmental origin of Health and Disease (DOHAD) hypothesis suggests that early life influences appear as the roots for placing the offspring at a high risk of perinatal mortality. Such early life exposures cause long lasting health effects worsening the quality of life and increased medical costs (Bilbo and Schwarz, 2009; Cottrell and Seckl, 2009; Bale et al., 2010; Haugen et al., 2014; Heindel et al., 2015; Wang et al., 2015). Environmental adversities such as malnutrition, immune challenges, stress, etc. during the pre-, neo-, and post-natal periods of development in experimental animals increase susceptibility to neuropsychiatric and neurodegenerative disorders during later life (Joles et al., 2010; Nestler and Hyman, 2010). Such observations in human have been reviewed by Pechtel and Pizzagalli (2011). Maternal diet and psychological factors appear as significant contributors to human health status evidenced by strong correlation between low socioeconomic conditions and compromised health (Stringhini et al., 2010; Monk et al., 2013) including risks for autism and schizophrenia (Woods, 1998; Krakowiak et al., 2012).

Malnutrition is a global burden most prevalent in the developing countries with Asia leading in the world. India, in particular has the highest number of stunted children (Müller and Krawinkel, 2005; Ahmed et al., 2012; Kerac et al., 2014; Raj et al., 2015). Protein malnutrition (PMN) during gestation cause developmental delays and is a major factor for intrauterine growth restriction and low birth weight in man (Connor, 2012) and mouse models (Whitaker et al., 2012). Nutrients are important in all phases of developing brain with respect to the neurons and glia development. However, nutritional inadequacy during prenatal periods have more severe effects as this period of development appears to be the most vulnerable and critical in primates (Antonow-Schlorke et al., 2011), rats (Alamy and Bengelloun, 2012; Prado and Dewey, 2014), and man (Aboud and Yousafzai, 2015). Thus, prenatal and/or early postnatal period of restriction has been the favorite model for major part of the available research literature. However, the impairments at levels of development, behavior, and cognitive function in children from malnourished mothers of underdeveloped countries is an outcome of a combinatorial effect of protein restriction during the pre-gestational and gestational periods along with the few years of postnatal life. The pre-gestational and gestational periods appear as the most critical windows for development with the former as a preparatory phase and latter a brain construction phase placing the developmental plan at a higher vulnerability. Effects of maternal PMN during pregnancy period on organ development can be directly related to functional outcomes in later life. Brain is programmed to grow more quickly than rest of the body and thus maternal nutrition during early and late gestation represents one of the major variables related directly to genesis, proliferation, differentiation, migration, and functional organization of various cell types in brain of human (Laus et al., 2011) and rat models (Alamy and Bengelloun, 2012). Clinical studies have established that malnutrition has a good correlation with alterations in neurodevelopment, physical growth parameters and brain structure (Grantham-McGregor and Baker-Henningham, 2005; Baker et al., 2013; Akitake et al., 2015).

Research on rodent models have established that PMN alters genesis, migration, differentiation, and plasticity of neurons during the critical stages of development (Gressens et al., 1997; Morgane et al., 2002; Alamy and Bengelloun, 2012; Godoy et al., 2013) and also affects the hippocamapal and hypothalamic neuronal proliferation and risk assessment behavioral outcomes in later life (Coupé et al., 2009; Torres et al., 2010; Reyes-Castro et al., 2011; Teicher et al., 2012). The alterations in processes like cell proliferations, differentiation, migration, synaptogenesis, and dendritic arborizations are central to poor neurocognitive development. Several studies have also shown biochemical changes in protein malnutrition (PMN) model involving specific enzymes and neurotransmitter systems in rats (Morgane et al., 2002; Steiger et al., 2003) and man (Contreras et al., 2010; de Souza et al., 2011; Zuker, 2015).

Experimental studies in rodents have established the consequences of malnutrition on brain development and behavior (Zhang et al., 2010; Reyes-Castro et al., 2012). Prenatal stress like maternal diet inadequacy, pollutants, toxicants, and distress have been reported to significantly affect the neuro-cognitive development (Bale et al., 2010; Glover, 2011; Sandman et al., 2011; Huang et al., 2013; Galler and Rabinowitz, 2014; Marques et al., 2014; Waber et al., 2014). Clinical studies have evidenced that undernourishment induced alterations are associated with delays in motor and cognitive functions like decreased IQ scores, impaired learning and memory, reduced social skills, and impaired school performance (Berkman et al., 2002; Kar et al., 2008; Hemb et al., 2010; Ghazi et al., 2014). Experimental studies have also established behavioral and cognitive impairments along with physical growth retardation and poor motor coordination in animal models of different stressors like maternal and early life infections, IUGR, micronutrient deficiency (Zhang et al., 2010; Mhillaj et al., 2015; Sarnyai et al., 2015). In rat (Petry et al., 2001) and human studies (Painter et al., 2005; Guedes, 2011) the malnourished offsprings presented a high morbidity in later life with drastic changes in learning and memory, social and other behaviors. They are predisposed toward a higher risk of developing neuropsychiatric disorders such as schizophrenia, depression, bipolar disorder, diabetes, obesity, hypertension, and glucose intolerancy.

Most of the previous investigators have studied the effects of gestational, lactational, or postnatal protein inadequacy (reviewed by Tolcos et al., 2011; Alamy and Bengelloun, 2012; Tomi et al., 2013; Marco et al., 2015; Wang et al., 2015). The limitation of such studies on rodent models has been the duration of malnutrition, i.e., the dams were exposed to a low protein diet for short durations, more or less than a week's time before pregnancy, thus modeling the gestational PMN only. Such maternal protein restriction for shorter durations immediately before pregnancy may not completely model the undernourished condition of females from low socio-economic groups in developing countries. Although, clinical relevant assessments show that pre-gestational malnutrition is a major component but the other periods are equally important and a combined effect of all critical windows would mimic the outcome similar to the malnourished population. Thus, to model the exact clinical conditions of IUGR in human females, an intra-generational protein restriction model of rats is expected to give a complete picture of the PMN induced behavioral and cognitive impairments in the offsprings. We have tried a new model of pre-gestational followed by gestational malnutrition by switching the naive SD females to a Low protein diet (8% protein) for 45 days before setting pregnancy. This level of pre-pregnancy protein reduction accurately emulates the human chronic protein malnourished status in the mother-to-be malnourished females of the developing countries. We investigated the low protein induced effects on early physical and reflex development and general abilities like locomoter activity, neuromuscular strength, spatial learning and memory as well as anxiety, and depression status. This study is the first of its kind to give a complete profile of the physical development, behavioral, and cognitive sequel of low protein F1 progeny with gender differences.

## Materials and methods

Nulliparous Sprague Dawley female rats (160–180 g, 2 month old) were housed under standard laboratory conditions in a 12 h light/dark cycle at 23 ± 2°C room temperature with *ad libitum* access to either of the two diets: (i) Low protein (LP, 8% protein, *n* = 8) or (ii) High protein (HP, 20% protein, *n* = 8) obtained from National Institute of Nutrition, Hyderabad, India as has been elaborated by Chaudhary et al. (2013). The animals were switched to the respective diets 45 days before conception and continued throughout gestational and lactational periods on the same diets. Timed pregnancies were set in the dams by a 4 h pairing with males. The stage of estrous with sperm was assessed by light microscopic examination of cells obtained from vaginal smears collected before 9 AM every morning and if positive, females were designated as Gestational Day 0 (GD0). Pregnant females were left undisturbed except for body weight measurements and cage cleaning twice a week. Timed pregnant females were observed carefully every 2 h on the expected days of delivery to mark the day of birth as postnatal day 0 (PND0). Following birth, each litter was culled to nine pups. Pups born to HP group mothers were weaned on PND21 but pups born to LP mothers were found to be weak and thus allowed to stay with the mothers with available LP diet. However, we did not analyse lactation time, weakness of the mother to produce milk and milk composition. The LP and HP pups were housed three per cage and maintained on the same diet until the termination of the experiment. The physical and reflex development was investigated between 9 and 12 AM for 15 consecutive days from PND2 to PND17 and behavior and cognitive tests were performed at 2, 3, and 6 months of postnatal age. Body weight of the pups was recorded daily till PND30. Following transcardial perfusion, on the specified experimental days, the brain was exposed, it was transected at the emergence of the spinal cord, transferred to a preweighed quantity of the fixative and then weighed to record the brain weight. This ensured that the brain is not drying during the procedure. All the experiments were performed with prior approval and in accordance with the Institutional Animal Ethics Committee of Jiwaji University, Gwalior.

### Evaluation of pregnant dam's behavior

Daily based food and water intake and body weight measurements (twice in a week) were performed for pregnant SD females between 9:00 and 11:00 AM in the morning.

### Neurological reflex and sensorimotor development in pups

A daily based investigation of pup reflex and other developmental landmarks was performed between 9:00 and 12:00 AM for 15 consecutive days (PND 2–17) until all pups in the litter reached the developmental landmarks with a score of 2. The tests were performed in the following chronological order surface righting reflex, negative geotaxis and cliff avoidance reflex. The developmental landmarks like pinna detachment, eye opening, and hair growth were evaluated until the pups reached the criterion. A daily based body weight measurement of pups was performed from PND 0 to 21.To avoid the stress, the separation time of pups from dams during the neurological reflex and sensorimotor investigation was restricted to less than 5 min. The sensorimotor reflexes were evaluated by recording the time the pup took to reach the criterion or complete the reflex and a score of 0, 1, or 2 was allotted (Fox, 1965; Tanaka et al., 2012).

### Surface righting reflex

Righting reflex was evaluated during PND 2-6 to measure motor function and coordination by placing the pups in a supine position on a plane surface and the time taken to adopt normal prone position by touching all the four paws with the plane surface was recorded within a cut-off time of 2 s. A score of “0” was allotted to the animals that couldn't perform within the cut-off time, “1” if reflex was achieved within 2 s and “2” if within 1 s.

### Negative geotaxis reflex

Considered diagnostic of vestibular and/or proprioceptive function, the negative geotaxis is an automatic stimulus where animals show a postural reaction by turning upright normally as a negative geotaxis reflex in an inclined plane test. A wooden board with rough surface was inclined at 25° and pups were placed on it with their snout downwards. The time taken to perform a 180° rotation was recorded with a cut-off time of 60 s. A score of “0” was allotted if the criterion was not achieved within the cut-off time, “1” if within 60 s and “2” if within 30 s.

### Cliff avoidance test

Animals display risk assessment behaviors by avoiding the cliffs and heights. Cliff avoidance reflex was evaluated in the pups from PND 3 to 11. The pups were placed on the edge of a wooden box with the forepaws and snout touching an imaginary line. The time taken by the animal to avoid the cliff and turn backwards was recorded. A score of “0” was allotted if the animal didn't show reflex within cut-off time, “1” if within time and “2” if reflex was shown with a backward turn.

### Open field test

The F1 generation rats (HP/LP) of 2, 3, and 6 month age group were individually placed in open field arena 43 × 43 × 22 cm high walls (Columbus Instruments, Ohio USA) with infrared beam detection system using Auto-track version 4.41 software. Animals were placed in the center of the dimly lit open field arena for 5 min to avoid the novelty induced exploration followed by recording the locomotor behavior for 20 min test session. The arena was cleaned with 70% ethanol between trials. The parameters of total distance traveled (DT), resting time (RT), stereotypic time (ST), and ambulatory time (AT) were recorded as indicative of spontaneous motor activity. Time in square analysis was performed from the individual open field track reports with increased time spent in the center zone considered as indicative of low anxiety.

### Elevated plus maze

To assess the anxiety and fear behaviors, elevated plus maze (EPM) consisting of a 40 (L) × 10 cm (W) arms raised 50 cm above the base was used. Of the four arms, two opposing arms were with 15 cm high walls (closed arms) and other two were without sidewalls (open arms). Before starting the test, the animal was placed in the central zone facing one of the open arms and exploratory behavior was recorded by camera placed above the apparatus attached to a computer with ANYmaze software (Columbus instruments, USA) for 2 min with three trials/animal. The time spent and number of entries to the open and closed arms were analyzed as measures of anxiety and fear.

### Forelimb gripstrength test

Grip strength meter (Columbus, USA) was employed for assessing neuromuscular function by sensing and recording the peak amount of force, the test animal applies in grasping specially designed pull bar assemblies. It consists of grid made up of steel wire measuring 76 × 250 mm and divided into rectangles of equal size providing a reliable surface for forelimb grip. This grasping device or platform was connected to dual sensor model located at a height of 9.5 cm, attached to a computer interface for measuring and saving the recorded data.

### Morris water maze test

Morris water maze (MWM) as an assay to test spatial learning and memory uses their ability of test animal to find a submerged platform in a tank (120 cm diameter with 50 cm high walls) filled with water (24 ± 1°C) with distinct spatial cues placed within the inner rim of the metallic tank. Days 1–3 constituted the learning phase in which animals were allowed to swim and locate the platform before 120 s. Those could not reach were guided to the platform location and retained there for 15 s. On the test day (4th day), the latency to locate the platform was recorded for three trials/animal. The platform was removed in the final trial to remove any chances of visual cue of the platform.

### Statistical analysis

All the statistical analysis was performed using the standard statistical software Sigma Stat 3.5. Values are expressed as mean ± standard error of the mean (SEM). Group comparisons were performed by One-way and Two-way analysis of variance (ANOVA), unpaired “*t*”-Test for inter group comparisons and *post-hoc* analysis for multiple comparison tests using Tukey's test and Holm-Sidak methods. The level of significance was set at a *P*-value of < 0.001 for highly significant and < 0.05 for significant.

## Results

**Figure 1:**
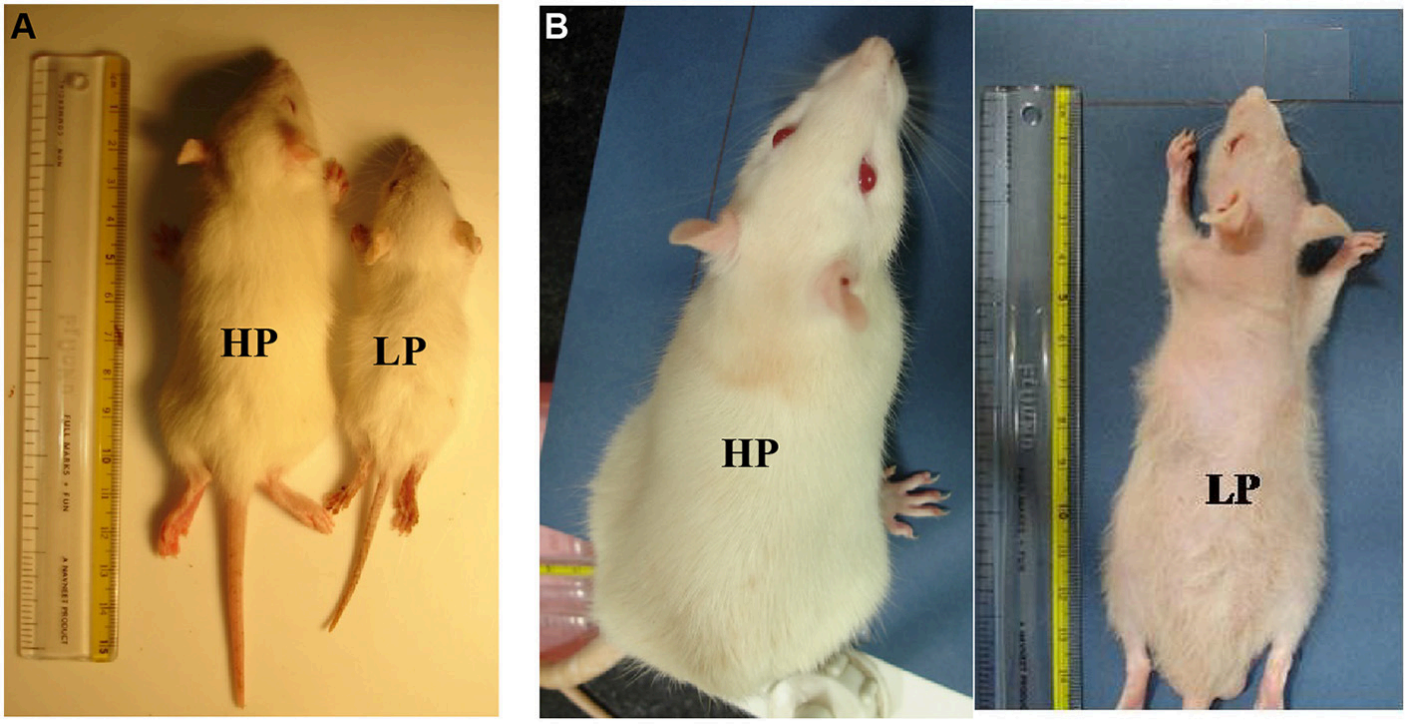
**Maternal PMN permanently compromises the body weight gain in later life: file photo of HP and LP group pups at the age of PND 21 (A) and 3 months (B) showing physical slowing and reduced body weight gain in LP F1 generation rats**. Loss of hair was a prominent feature at 3 months of age in LP rats.

No significant LP diet induced effects were observed on litter size and sex ratio at birth (data not shown). A greater mortality index was noticed in LP group animals (pups/adults) as compared to the HP controls. During the pre-weaning period (PND 0–21), the mortality index was 40% in LP group and 10% in HP group. The mortality was still higher, i.e., 55% in LP group till last study time point. No significant differences were found in parameters like pinna detachment, eye opening and hair growth (data no shown) between LP and HP pups. However, the LP males presented a complete fur loss at 2 months of postnatal age, which started reappearing by the end of 3rd month (Figure 1A).

### Early life protein malnutrition permanently compromises body weight gain in LP F1 progeny

**Figure 2:**
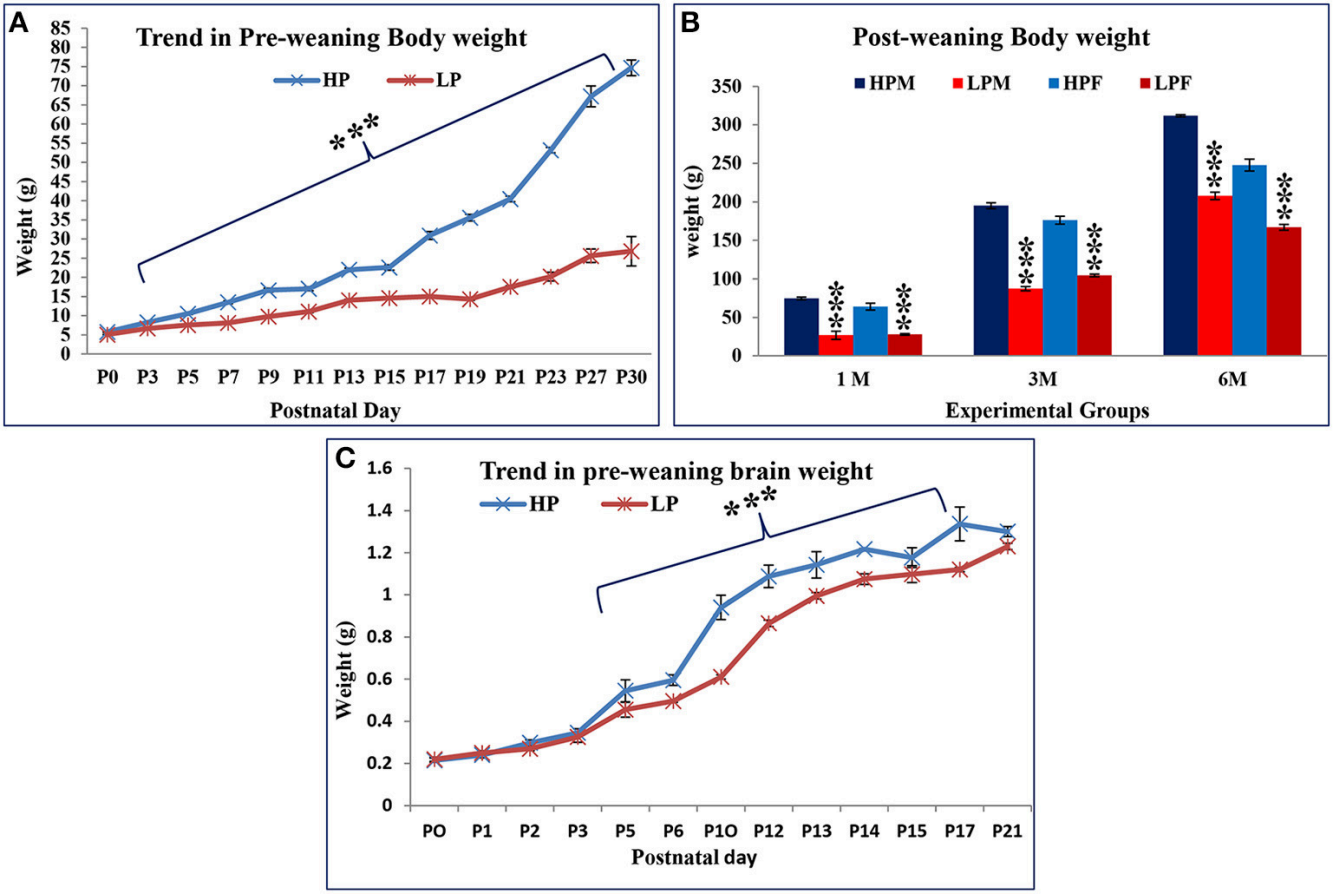
**Lactational but not gestational protein malnutrition results in decreased body and brain weight gain in F1 generation: A daily based pre-weaning body weight gain trend in HP and LP group pups with advancing postnatal age revealed a decelerated growth in the LP F1 pups**. Surprisingly, no significant difference was noticed at birth (PND 0). However, the body weight difference was highly significant from PND 3 to 30 **(A)** and even at maturity **(B)**. Graph **(C)** shows the gross brain weight of LP and HP group animals on some representative days. By PND 5 onwards, the gain in brain weight was consistently and significantly lower in LP animals and continued till the last study time-point. Data is presented as Mean body/brain weight in grams ± SE. ^***^P < 0.001.

The F1 progeny from LP dams showed a definite compromise in physical growth in terms of reduced body and brain weight gain with age (Figures 1, 2A–C). One-way ANOVA revealed significant effects of LP diet on the body weight gain [*F*_(1, 39)_ = 138.2, *p* < 0.001]. The *post*-*hoc* comparisons revealed that LP litters weighed significantly less than HP controls on all days from PND 1 to 21 (*p* < 0.001), although there was no significant difference at birth, i.e., PND0 (Figure 2A). Even at maturity and thereafter i.e., 2, 3, and 6 months of age, the LP rats showed persistently and significantly lower body weight as compared to their age matched HP rats (*p* < 0.001, Figure 2B). In addition, there was a gender dependant difference in the HP animals, with males displaying significantly higher body weight as compared to females [*t*_(21)_ = 7.87 = *p* < 0.05]. Although no significant sex related difference in average body weight amongst the LP group rats was observed till weaning period. Surprisingly at 3 months of age, the females significantly outweighed their respective age matched males [*t*_(21)_ = 9.14 = *p* < 0.05], whereas males outweighed females by 6 month (Figure 2B).

The average brain weight showed a similar trend as body weight in both HP and LP F1 generation pups (Figure 2C). The average brain weight of LP group animals always lagged as compared to respective HP controls, but a significant difference was noticed from PND 5 till last brain harvested time point (*p* < 0.001).

### Physical and neuro-motor development in F1 pups along advancing postnatal age

The impact of maternal protein deprivation on reflex ontogeny was evaluated by employing a battery of physical development and neurological tests. A gender specific data (percent mean scores) for specific reflex attainment against postnatal day is presented in Tables 1A–C. A general significant delay in reflex attainment and poor performance in terms of score was registered in LP F1 pups.

**Table 1:** **Score frequencies as percent animals reaching the criterion for neuro-reflex development in F1 generation SD rats (neonatal to pre-weaning period), born to HP, and protein restricted dams (LP)**.

**A: SURFACE RIGHTING REFLEX (PND3–PND7)**													
**LP F1 FEMALE**													
**Score**			**PND3**			**PND5**				**PND6**			**PND7**
			Percent score frequency										
0			23.8			0				0			0
1			47.61			28.57				4.76			0
2			28.57			71.4				95.23			**100**
**HP F1 FEMALE**													
**Score**			**PND3**			**PND5**				**PND6**			**PND7**
0			28			16				4			0
1			16			32				16			0
2			56			52				80			**100**
**LP F1 MALE**													
**Score**			**PND3**			**PND5**				**PND6**			**PND7**
0			23.07			0				0			0
1			61.53			15.38				15.38			0
2			15.38			84.61				84.61			**100**
**HP F1 MALE**													
**Score**			**PND3**			**PND5**				**PND6**			**PND7**
0			35.29			5.88				5.88			0
1			17.64			23.52				11.76			0
2			47.05			70.58				82.35			**100**
**B: INCLINED PLANE TEST (PND2–PND10)**													
**LP F1 FEMALE**													
**Score**		**PND2**		**PND3**			**PND5**		**PND7**			PND9^**^	
		Percent score frequency											
0		78.78		60.6			34.28		5.71			0	
1		21.21		24.24			25.71		22.85			0	
2		0		15.15			40		71.42			**100**	
**HP F1 FEMALE**													
**Score**		**PND2**		**PND3**			**PND5**		**PND7**				
0		33.33		74.35			7.14		0				
1		33.33		7.69			21.42		0				
2		33.33		12.82			71.42		**100**				
**LP F1 MALE**													
**Score**		**PND2**		**PND3**			**PND5**		**PND7**			**PND9**	
0		100		93.75			75		20			0	
1		0		6.25			12.5		6.66			0	
2		0		0			12.5		73.33			**100**	
**HP F1 MALE**													
**Score**		**PND2**		**PND3**			**PND5**		**PND7**				
0		82.6		82.6			21.73		0				
1		13.04		13.04			34.78		0				
2		4.34		4.34			43.47		**100**				
**C: CLIFF AVOIDANCE (PND3–PND11)**													
**LP F1 FEMALE**													
**Score**	**PND3**		**PND5**		**PND7**	**PND9**		**PND11**			PND12^**^		
		Percent score frequency											
0	93.1		72.41		37.93	31.03		7.69			0		
1	6.89		20.68		27.58	17.24		38.46			0		
2	0		6.89		31.03	68.96		53.84			**100**		
**HP F1 FEMALE**													
**Score**	**PND3**		**PND5**		**PND7**								
0	44.44		44.44		0								
1	48.14		37.03		0								
2	7.4		18.51		**100**								
**LP F1 MALE**													
**Score**	**PND3**		**PND5**		**PND7**	**PND9**		**PND11**			PND12^**^		
0	100		76.47		41.17	7.69		10			0		
1	0		23.52		35.29	30.76		30			0		
2	0		0		23.52	30		60			**100**		
**HP F1 MALE**													
**Score**	**PND3**		**PND5**		**PND7**								
0	64.7		16.66		0								
1	35.29		50		0								
2	0		33.33		**100**								

Each value represents the score frequency (%), percent of the total pups achieving the criterion with respective score. A, Surface Righting Reflex (PND3–PND7); B, Inclined plane test (PND2–PND10); C, Cliff avoidance (PND3–PND11).

** P < 0.001; Bold values indicate 100% achievement of the reflex.

**Figure 3:**
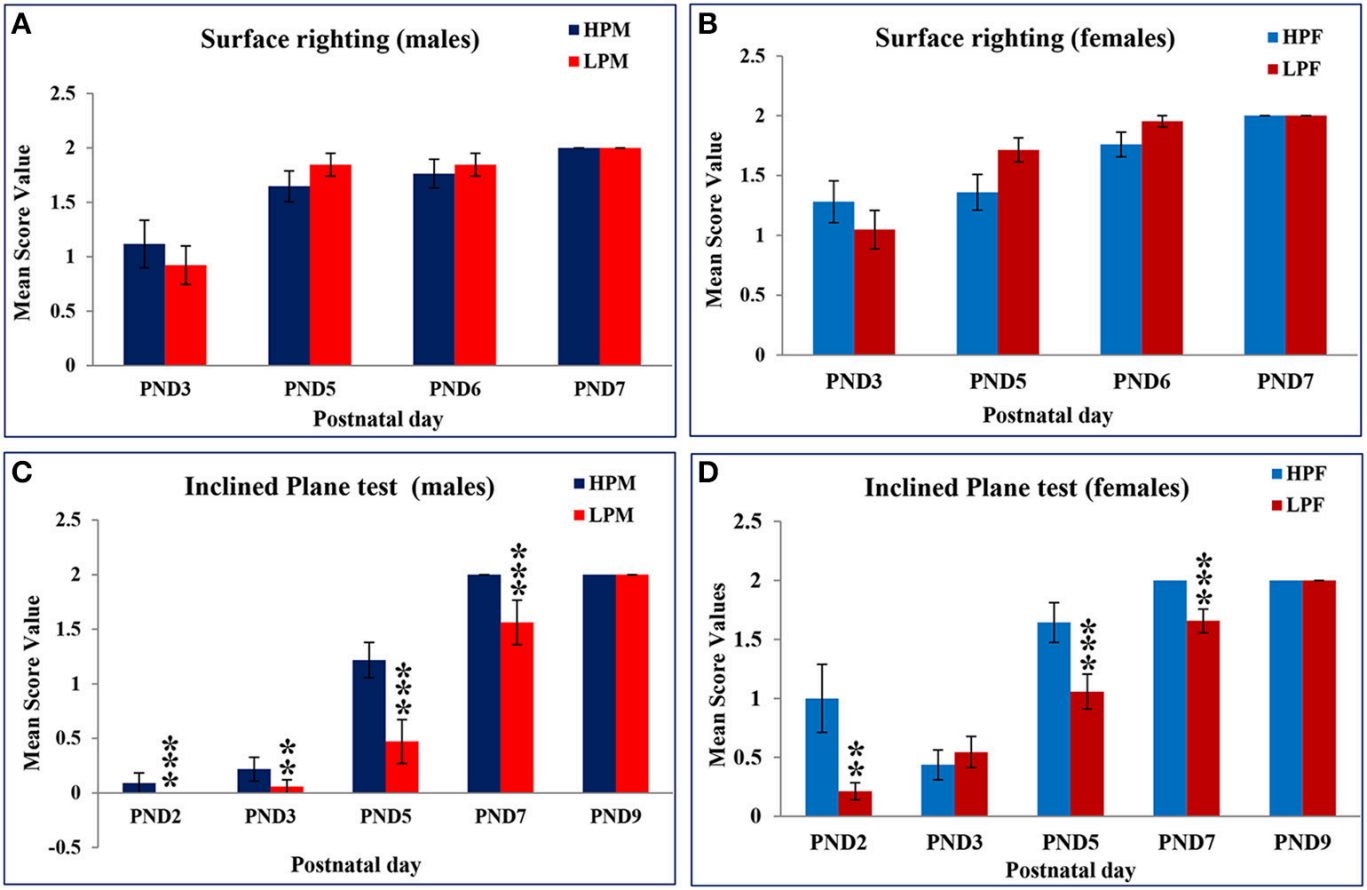
**Maternal PMN delays neurodevelopment and performance in F1 generation rats: Graphs showing the Surface righting reflex in HP and LP group males (A) and Females (B) as Mean score value against postnatal day**. A complete surface righting reflex with a score of 2 was attained by PND 6 in both HP and LP Pups with no significant sex related difference. Inclined Plane test results in HP and LP F1 males **(C)** and Females **(D)** as mean score value against postnatal day for 100% attainment of the reflex reveal a significant effect of pre-gestational protein malnutrition on the reflex maturation, with HP F1 pups presenting the negative geotaxis response significantly earlier and better in terms of scoring. Data is presented as Mean Score Value ± SE. ^**^P < 0.01, ^***^P < 0.001.

Data from behavioral developmental parameters revealed no significant difference in surface righting reflex between the LP and HP F1generation pups. This reflex was initiated at PND 2 in both the LP and HP pups. However, the expression in LP pups increased quickly to reach maturation (100%) by PND 5, with a score of 2 while in HP a 100% with 2 score criteria was achieved only at PND 7. Amongst group analysis revealed that LP F1 females elicited this reflex better in terms of performance (score frequency) followed by LP males as compared to HP F1 Pups (Table 1A; Figures 3A,B).

A Two-way ANOVA revealed a significant effect of maternal protein malnutrition on inclined plane test [*F*_(1, 30)_= 8.101, *p* ≤ 0.001] and cliff avoidance [*F*_(1, 30)_ = 11.034, *p* ≤ 0.001] indicating a delay in the day of apparition of these neurological reflexes in LP group animals. The Inclined plane test (negative geotaxis reflex) revealed a significant difference in expression and performance against postnatal day in LP group animals as compared to respective age matched HP controls. This precocious reflex was expressed at PND 2 in all pups except for LP males where it appeared by PND 3 (Figures 3C,D). One-way ANOVA revealed a significant difference in LP males in the time taken to reorient themselves at PND 2 [*F*_(3, 156)_ = 11.816, *p* ≤ 0.001], PND 3 [*F*_(3, 156)_ = 16.214, *p* ≤ 0.001], PND 5 [*F*_(3, 156)_ = 9.012, *p* ≤ 0.001], and PND 7 [*F*_(3, 156)_= 10.826, *p* ≤ 0.001] as compared to the HP males (Table 1B, Figures 3C,D). The LP males displayed difficulty and took more time to reorient themselves as compared to the LP females. Although, HP females took less time to complete the reflex than HP males, no significant sex related difference was observed in score frequencies. The HP pups attained 100% reflex by PND 7, whereas, the LP pups presented a delay of 2 days in attaining 100% negative geotaxis response (Table 1B).

**Figure 4:**
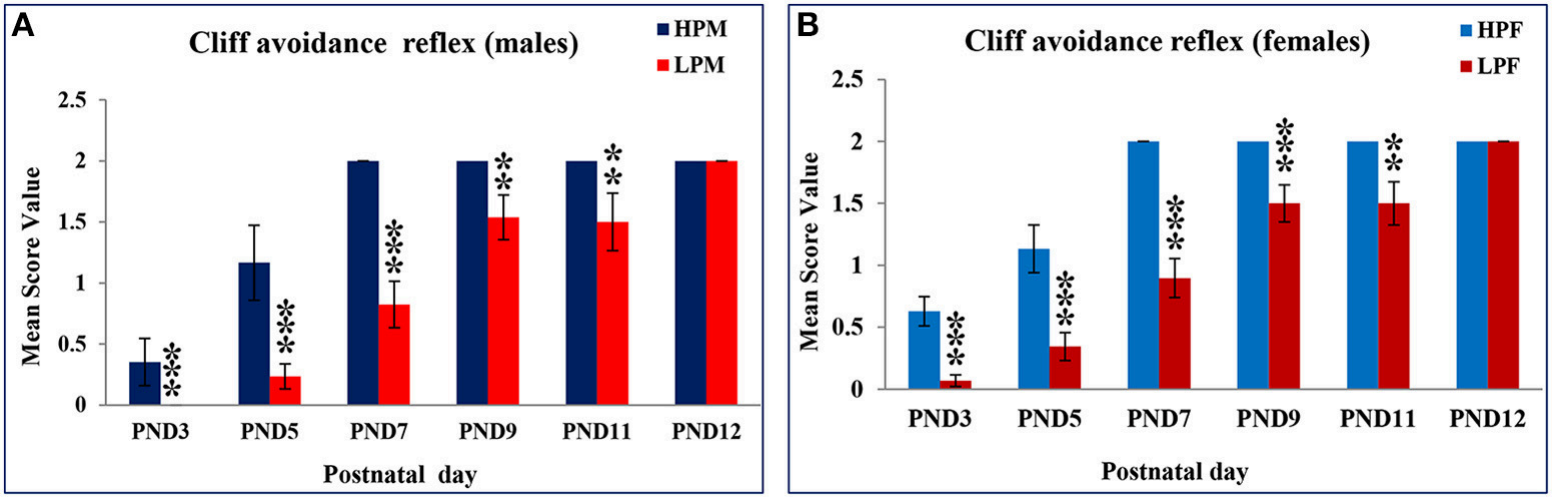
**Maternal PMN significantly delays cliff avoidance reflex in F1 generation pups: Graphs showing the Cliff avoidance reflex data as mean score value in HP and LP group males (A) and Females (B) at representative postnatal days**. HP pups attained this reflex by PND 7 with no sex dependent difference in performance on score levels and reflex completion. A statistically significant delay of 5 day was recorded in LP group pups as the reflex maturation was attained by PND 12. Data is presented as Mean Score Value ± SE. ^**^P < 0.01, ^***^P < 0.001.

LP diet also induced a delay in cliff avoidance reflex expression and poor performance. HP pups of both sex took less time to display avoidance reflex and 100% of the reflex with a score of 2 was attained by PND 7 (Table 1C, Figures 4A,B). Data of score frequency revealed that LP pups required more time and displayed significant delay of 5 days in 100% reflex completion with a score of 2 at PND 12 (Table 1C). A significant difference was found in the time taken to elicit cliff avoidance reflex in LP males at PND 3 [*F*_(3, 156)_ = 18.016, *p* ≤ 0.001] and PND 5 [*F*_(3, 156)_ = 16.237, *p* ≤ 0.001].

### Intra-generational protein malnutrition induces hyperactivity and low anxious behavior in open field test

**Figure 5:**
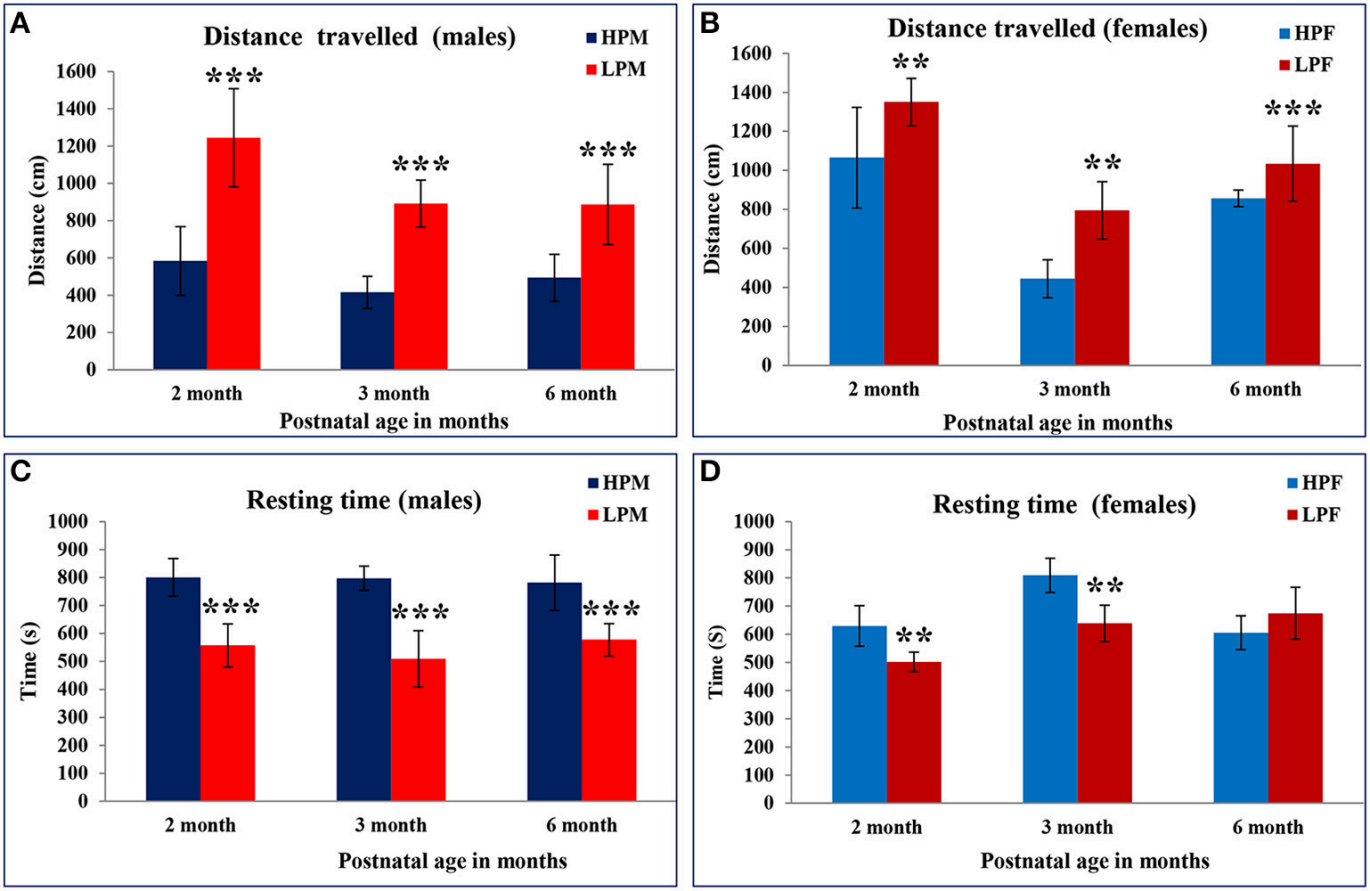
**Hyperactivity and anxiety like behavior displayed by LP F1 generation animals: Bar graphs representing the distance traveled (A,B) and resting time (C,D) in Open field test of HP and LP group male and female rats at 2, 3, and 6 months of postnatal age**. LP F1 animals showed hyperactivity as evident by significantly increased distance traveled and less resting time as compared to HP group animals. Data is presented as Mean ± SE. ^**^P < 0.01, ^***^P < 0.001.

We investigated the intra-generational low protein effects on the anxiety related behaviors in Open field. Low protein group animals were showing hyperactivity with significantly increased distance traveled at all postnatal age time points studied i.e., 2 [*F*_(3, 68)_ = 7.398, *p* ≤ 0.001], 3 [*F*_(3, 68)_ = 8.374, *p* ≤ 0.001], and 6 month [*F*_(3, 68)_ = 10.787, *p* ≤ 0.001]. There was a main effect of diet and sex on the total distance (DT) moved in the open field [*F*_(1, 22)_ = 7.334, *p* ≤ 0.001; Figures 5A,B].

**Figure 6:**
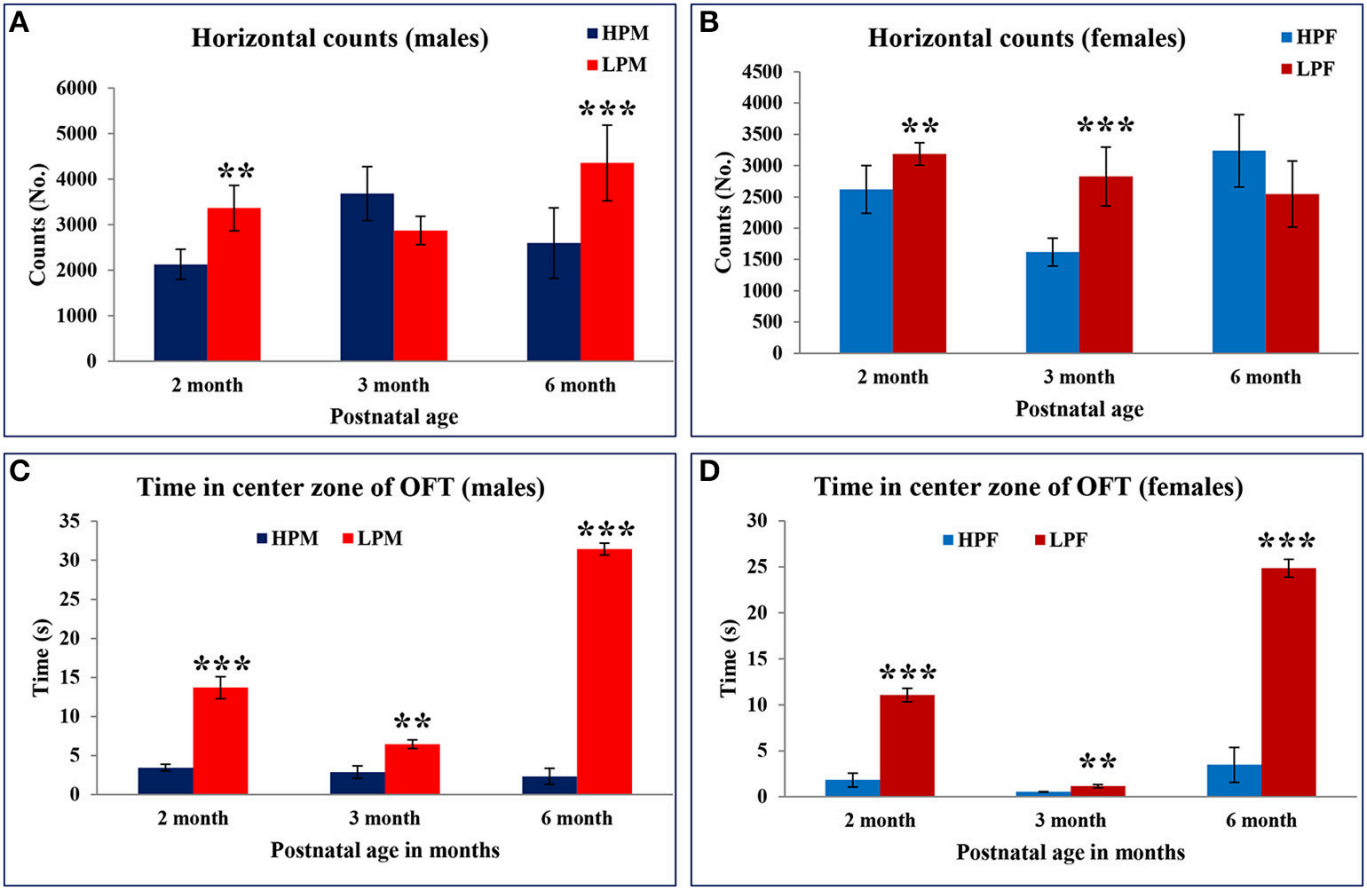
**Maternal protein malnutrition induces hyperactivity and low basal anxiety in F1 generation pups: Graphical representation of horizontal counts (A,B) and time spent in center zone (C,D) of the Open field test in HP and LP F1 generation rats at 2, 3, and 6 months of age as a measure of the anxiety**. Horizontal counts in open field estimate the basal activity of the animal with increased horizontal counts as a measure of the hyperactive behavior. LP males at the age of 2 and 6 months displayed increased horizontal activity. The behavioral bias in response at 3 months can be correlated to the sexual maturity and associated HPA hormonal axis. Time in square analysis revealed low basal anxiety levels and reduced habituation in LP group animals at 2 and 6 months of postnatal age irrespective of sex as compared to the HP controls. Data is presented as Number of counts and Time spent in seconds (Mean ± SE). ^**^P < 0.01, ^***^P < 0.001.

**Figure 7:**
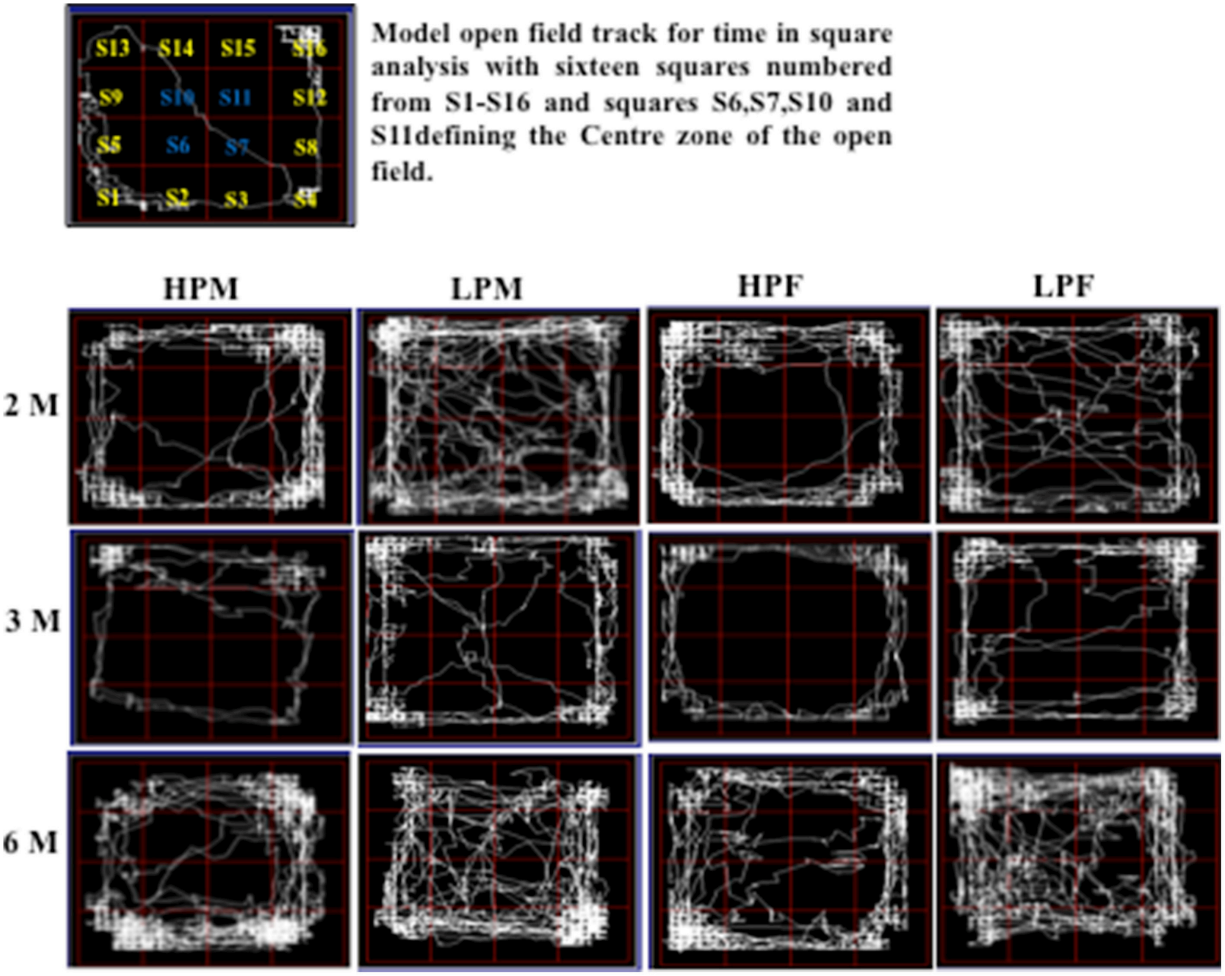
**Track reports of open field test: Representative open field activity tracks of 2, 3, and 6 month old rats of HPM (HP F1 males), LPM (LP F1 males), HPF (HP F1 females), and LPF (LP F1 females) groups**. The time in square analysis reveal low basal anxiety and reduced habituation in LP group animals irrespective of sex with significantly more time spent in center zone as compared to HP age matched controls.

As a result of the hyperactivity in the LP group animals, the resting time duration was significantly reduced at 2 [*F*_(3, 68)_ = 5.816, *p* ≤ 0.005], 3 [*F*_(3, 68)_ = 7.576, *p* ≤ 0.001], and 6 months [*F*_(3, 68)_ = 8.491, *p* ≤ 0.001] of age as compared to the respective HP group animals (Figures 5C,D). A similar trend was noticed for the horizontal counts (HC) as a measure of horizontal activity which was significantly high in LP group animals irrespective of sex at 2 [*F*_(3, 68)_ = 7.296, *p* ≤ 0.005], 3 [*F*_(3, 68)_ = 7.576, *p* ≤ 0.001], and 6 month [*F*_(3, 68)_ = 9.091, *p* ≤ 0.001] of age as compared to the HP controls (Figures 6A,B). In general the females significantly moved greater distance as compared to males at all the study time-points (Figure 7).

LP group animals irrespective of sex showed a significantly lower latency to enter the center zone. Time in square analysis revealed LP group animals spent significantly more time in the center zone at 2 [*F*_(3, 68)_ = 7.308, *p* ≤ 0.001], 3 [*F*_(3, 68)_ = 8.307, *p* ≤ 0.001], and 6 months [*F*_(3, 68)_ = 10.763, *p* ≤ 0.001] of postnatal age with respect to age matched HP controls (Figures 6C,D, 7).

**Figure 8:**
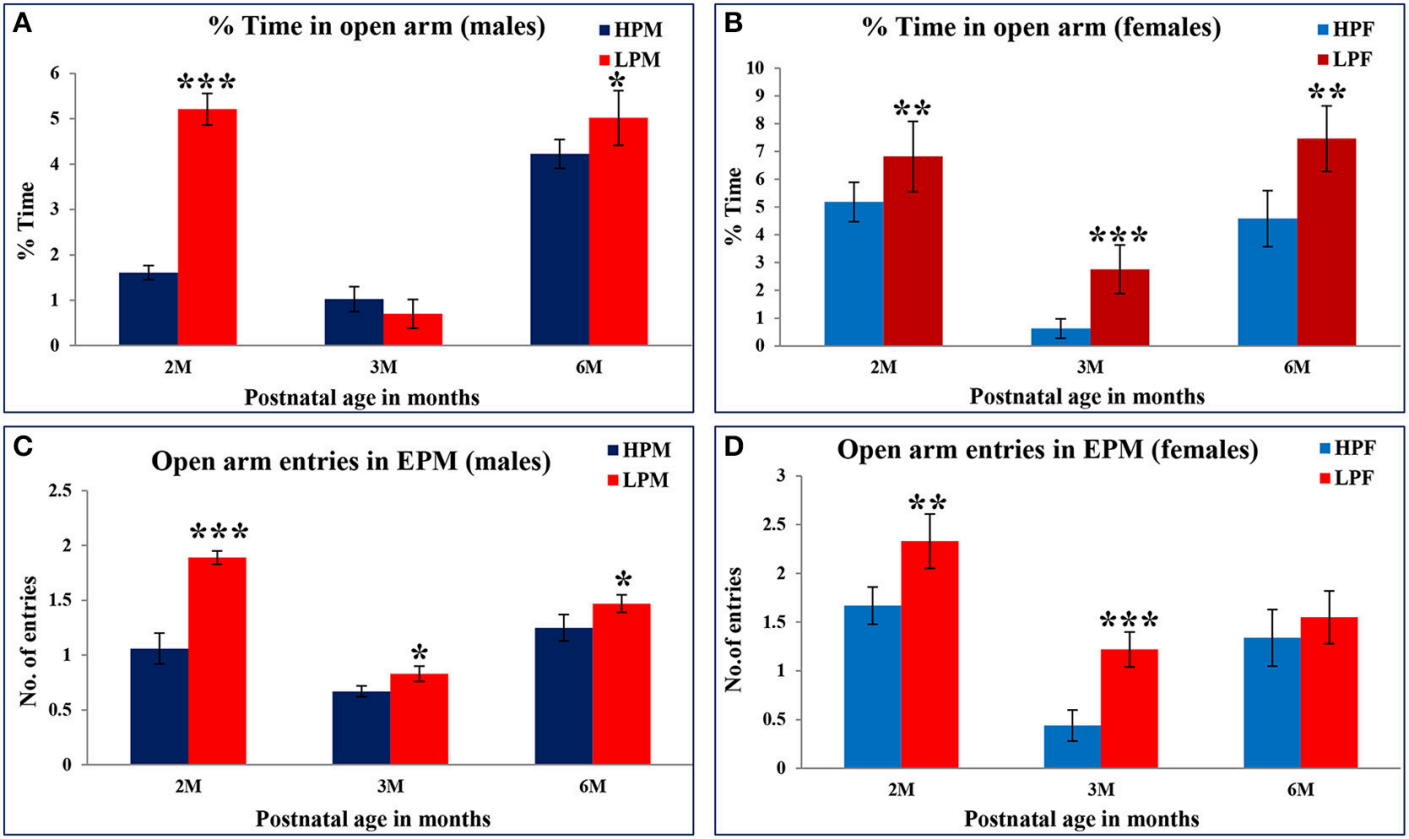
**Percent time spent in open arms of Elevated Plus maze as a measure of anxiety level in rodents: Bar graphs representing the percent time spent in open arms at 2, 3, and 6 months of postnatal age**. A significant increase in both percent time in open arms and open arm entries in LP group animals at 2, 3, and 6 month postnatal age is an indicative of low anxiety and low fear behavior further validating the results from open field test. Data is presented as % time spent (Mean ± SE) (males, **A**; females, **B**) and open arm entries (males, **C**; females, **D**). ^*^P < 0.05, ^**^P < 0.01, ^***^P < 0.001.

Elevated plus maze test (EPM) was employed to investigate the LP diet induced effects on anxiety level in LP or HP F1 generation rats. Data analysis from EPM test revealed that irrespective of sex, LP animals of all age groups spent significantly more time in open arms [percent open arm time 2 [*F*_(3, 68)_ = 6.119, *p* ≤ 0.001], 3 [*F*_(3, 68)_ = 6.391, *p* ≤ 0.001], and 6 months [*F*_(3, 68)_ = 10.095, *p* ≤ 0.001] with an increased tendency to explore the open ends (Figures 8A–D). During the test interval a large proportion of LP group animals fell off from the open arm ends evidencing hyperactivity and less fear behavior. LP group animals showed a similar performance in the open field test as well suggesting LP diet induced hyperactivity and less anxious/low fear like behavioral traits.

### Maternal protein malnutrition results in reduced neuromuscular strength

**Figure 9:**
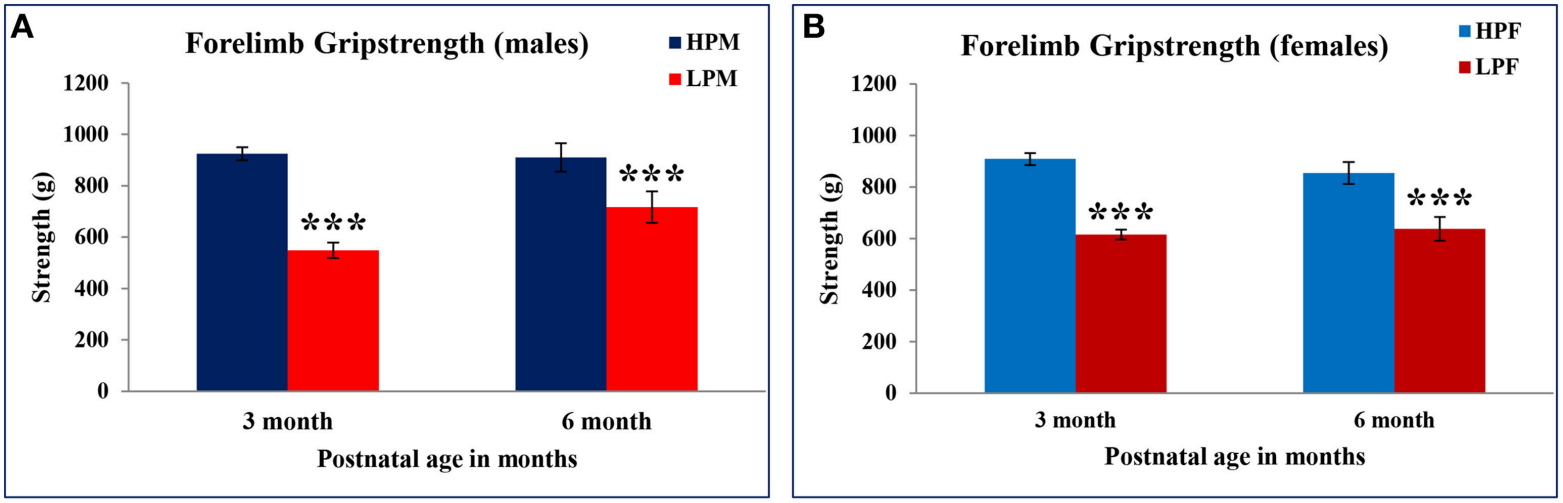
**Maternal PMN induces poor neuromuscular strength in F1 generation: Bar graph showing Forelimb gripstrength in HP and LP group males (A) and females (B) subjected to gripstrength test at 3 and 6 months of postnatal age**. A significantly poor neuromuscular strength was recorded in LP group animals irrespective of sex at 3 and 6 months of postnatal age. Data is presented as Mean ± SE. ^***^P < 0.001.

Forelimb gripstrength test was employed to evaluate LP diet induced effects on neuromuscular strength. LP F1 rats showed significantly reduced neuromuscular strength as compared to age matched HP controls at 3 [*F*_(3, 68)_ = 10.886, *p* ≤ 0.001] and 6 months [*F*_(3, 68)_ = 9.262, *p* ≤ 0.001] of postnatal age (Figures 9A,B). A greater sensitivity to LP diet effects toward males was seen with LP males displaying significantly reduced forelimb gripstrength at 3 months [*F*_(3, 68)_ = 5.886, *p* ≤ 0.005] as compared to LP females. Overall, the LP group rats presented laziness to grasp and required more attempts for successful recordings of forelimb gripstrength.

### Morris water maze (MWM) test reveals impaired spatial learning and memory in LP F1 rats

**Figure 10:**
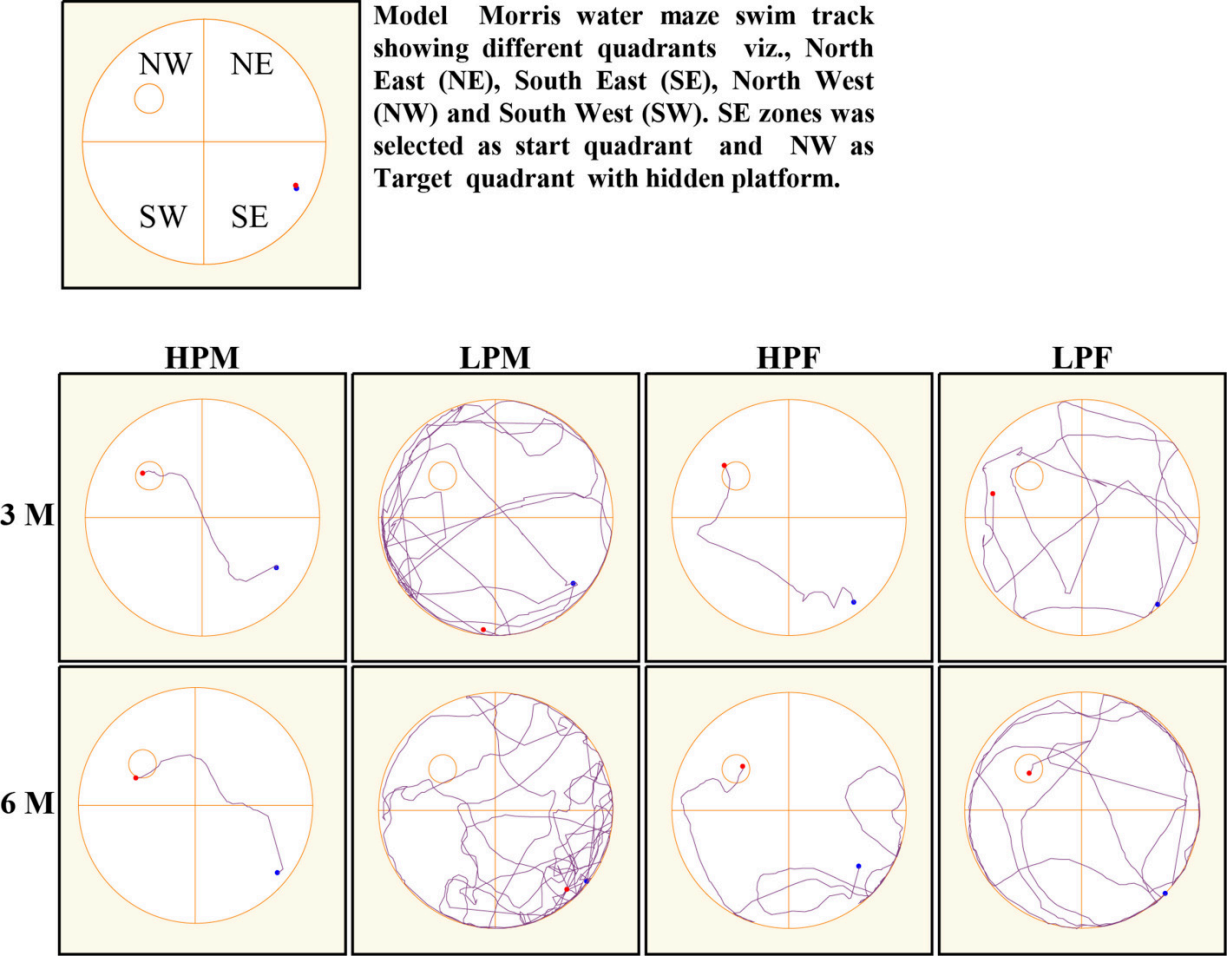
**Morris water maze (MWM) tracks indicate poor spatial learning and memory: Representative swim tracks of HP and LP F1 animals at 3 and 6 months of postnatal age**. The swim tracks represent poor spatial learning and memory in LP group animals with severe effects manifested in LP males. HP group animals swam less, presented good memory with a high path efficiency.

**Figure 11:**
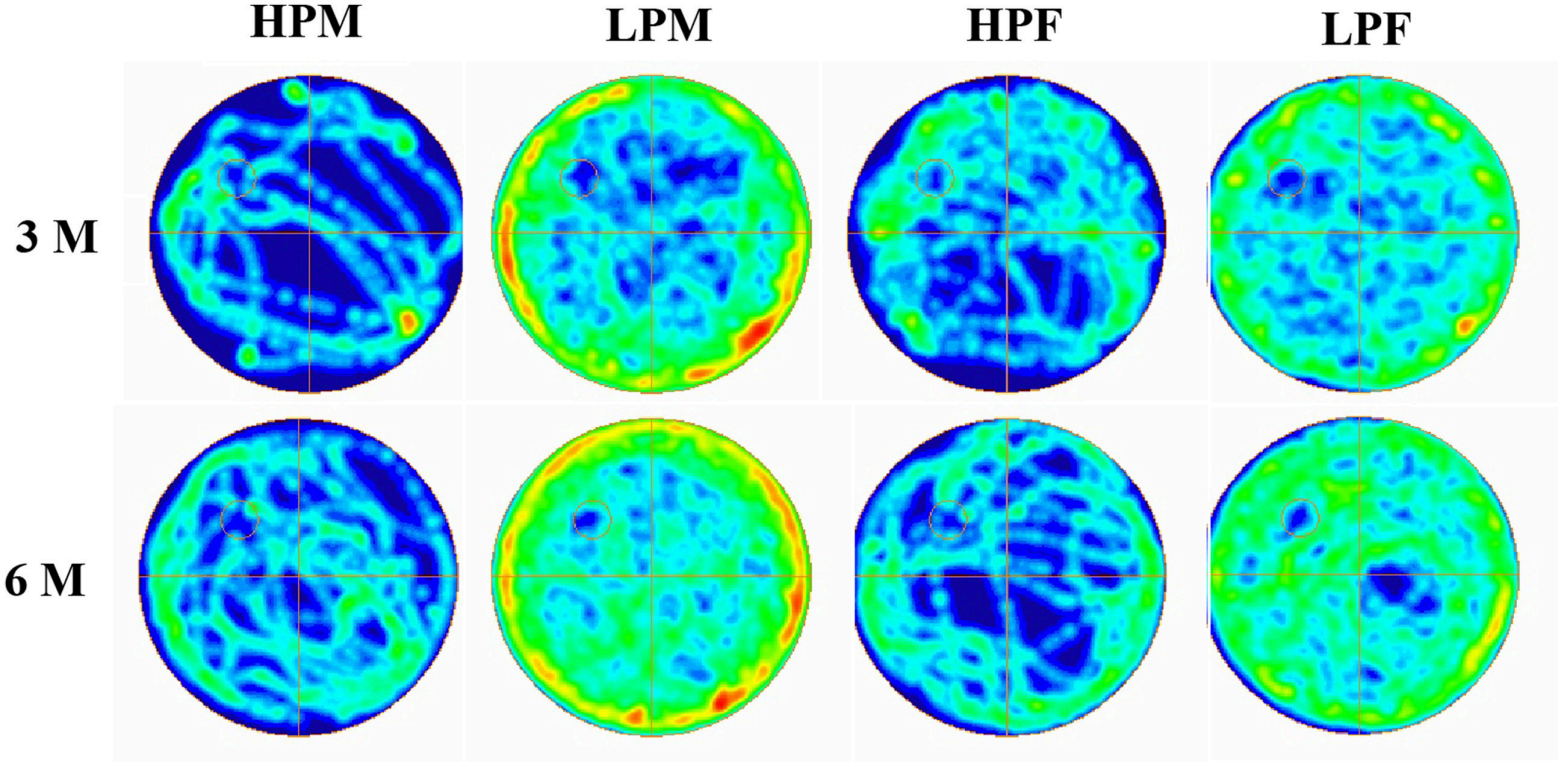
**Group occupancy plots in MWM test reveal hyperactivity and learning and memory impairments in LP animals**. ANYMaze Software generated group occupancy plots for activity in MWM test reveals efficient learning in HP males and females at 3 months whereas LP males and Females show more swim activity reflecting poor spatial memory. Spatial learning and memory deficits with hyperactivity in MWM task in LP group animals is evident in 6 month age group.

**Figure 12:**
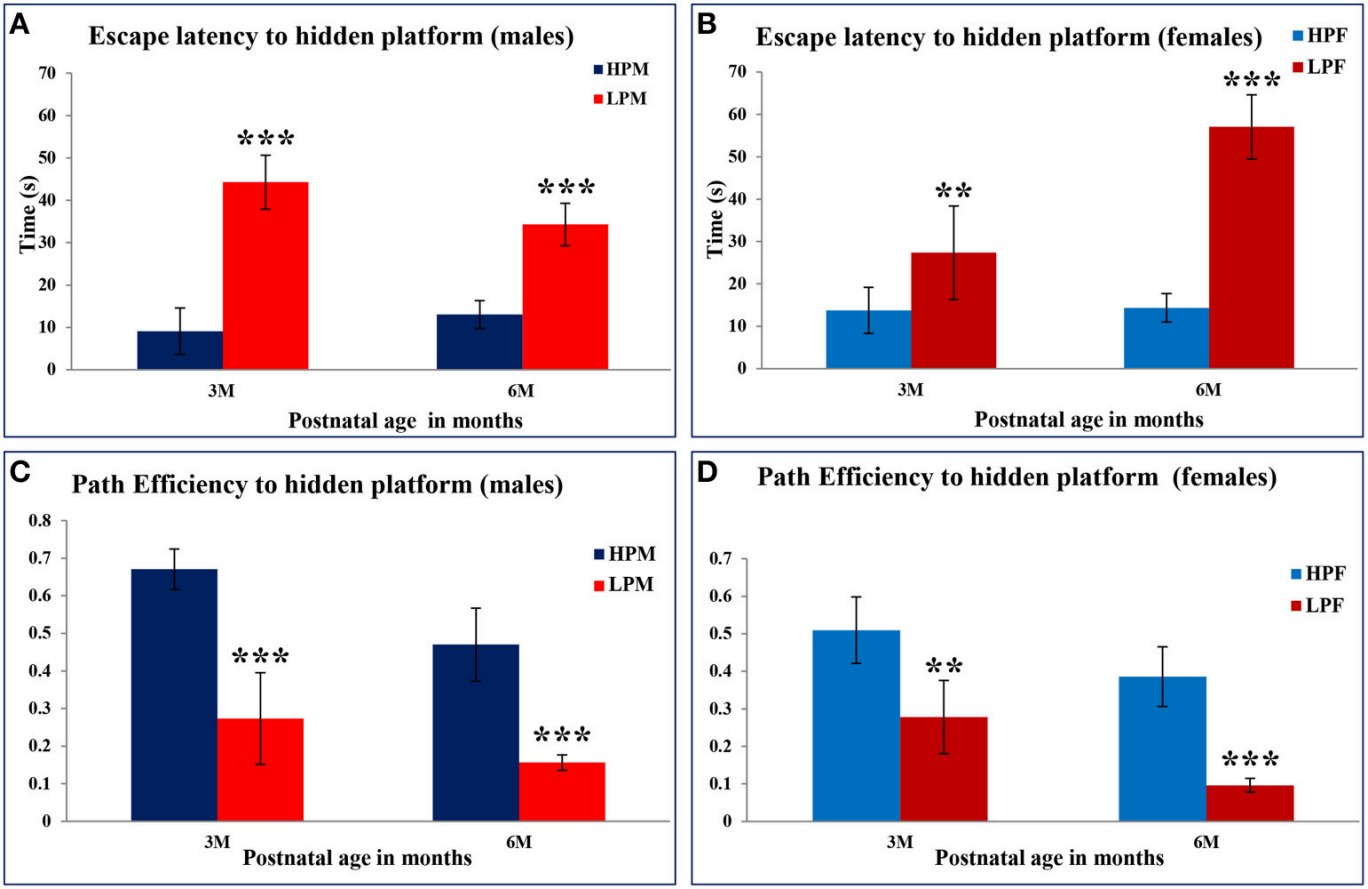
**Maternal PMN leads to impaired spatial learning and memory in later life: Bar graphs representing the latency to locate the hidden platform (A,B) and path efficiency (C,D) in MWM test at 2, 3, and 6 months of postnatal age in HP and LP group animals**. Data from the MWM test reveals a significantly high latency in the LP group animals to locate the hidden platform. A significant better path efficiency was noticed in HP group animals with males displaying high efficiency as compared to females well correlating with the natural tendency of females loving to swim and float. Data is presented as Mean ± SE. ^**^P < 0.01, ^***^P < 0.001.

MWM test was used to evaluate the impact of intra-generational protein malnutrition on spatial learning and memory function at 3 and 6 months of postnatal age. No MWM test was performed at 2 months of age as LP animals couldn't swim well. The results of the retention trials of the MWM test in LP and HP group animals indicated the LP diet induced spatial learning and memory deficits (Figure 10). The path efficiency for the training blocks was significantly low in the LP group rats as compared to the age matched HP controls both at 3 [*F*_(3, 92)_ = 6.806, *p* ≤ 0.005] and 6 month [*F*_(3, 92)_ = 5.217, *p* ≤ 0.005] of postnatal age (Figures 11, 12A–D).

Group occupancy plots from MWM test revealed that LP group rats took progressively more time to locate the hidden platform during the retention trials on the 4th Day (Figure 11). In addition to this, a significantly high latency to locate the platform was also noticed in LP group animals with gender specific effects, where females outperformed males (Figure 11). The hyperactivity with poor memory retention in LP males as evident from group occupancy plots suggest the severe LP induced effects in males at 3 and 6 months of postnatal age (Figure 11). Swim track analysis revealed that LP group animals swam for longer durations and presented jumbled tracks with most time spent out of the target quadrant as compared to controls, depicting continued and persistent spatial cognitive deficits (Figures 10, 11).

## Discussion

CNS development *in-utero* is critically determined by maternal nutrition especially protein component, deprivation of which culminates at aberrant neurodevelopment. The degree of malformation depends on the timing and magnitude of the insult and the effects are exacerbated if it occurs during critical developmental windows. Rodent brain development has been correlated to the brain growth spurt, that extends from the human second trimester to first 2 years of postnatal life, corresponding to the critical and vulnerable time involving neurogenic and gliogenic events (Dobbing, 1968; Mendez and Adair, 1999; Patro et al., 2015). Maternal diet being determined by the pregestational and gestational diets, its transmission through the placenta determines the fetal metabolism. Protein deprivation in rat maternal diet have been variously reported to cause permanent deleterious effects on the developmental profile of the developing fetus (Almeida et al., 1996; Gressens et al., 1997; Alamy et al., 2005; Alamy and Bengelloun, 2012; Reyes-Castro et al., 2012).

Protein restriction during development interferes with the neocortical morpho-functional and sub-cortical synaptic organization and affects the biochemical, behavioral, and electrophysiological consequences of individual, thus limiting cognitive abilities and other behavioral patterns. The resulting altered fetal programming impairs the physiology, organ development, function, maturation, hormonal profile, and pre-disposition to metabolic, mental and cognitive disorders in rats (Levay et al., 2010; Del Arco et al., 2011; Zhang et al., 2012; Marques et al., 2014) and human (Kar et al., 2008; Waber et al., 2014; Georgieff, 2015; reviewed by Galler and Rabinowitz, 2014) Despite extensive literature evidencing the detrimental effects of maternal protein malnutrition on behavioral and cognitive deficits in animal models, most of the studies have followed short restriction protocols either modeling the gestational or early gestational malnutrition along with lactational periods (Glover, 2011; Sandman et al., 2011; Alamy and Bengelloun, 2012; Kerac et al., 2014). Such short protocols of protein restriction don't completely emulate the clinical conditions of females in developing and underdeveloped countries. In this study we have tried a model of pre-gestational, gestational, lactational, and postnatal protein deprivation by switching naive SD dams on LP (8% protein) diet 45 days prior to mating and continued through pregnancy. The malnutrition regimen was thus not limited solely to pre-gestational and gestational periods, pups born to LP group dams were maintained on the same diet post-weaningly and throughout the study period. The present paper aimed to investigate the effects of intra-generational effects of protein malnutrition on the physical development, reflex ontogeny, behavioral and cognitive profile of the F1 generation.

With the well-established concept of low protein induced IUGR, some earlier studies in rats have reported accelerated growth in maternally malnourished fetuses until the last phase of gestation at which it becomes restricted resulting in a fetus with normal body weight but with constrained truncal growth (Langley-Evans et al., 1996, 2006) which find bearing on human life (Gluckman and Hanson, 2004). However, other rodent studies have observed significant differences in organ weights of fetuses pre-exposed to protein malnourishment during pregnancy (Alamy and Bengelloun, 2012). In line with earlier studies, we also noticed a lesser increase in the bodyweight gain of low protein fed dams in the last week of pregnancy and compromised later life body weight gain in LP F1 rat pups (Lukoyanov and Andrade, 2000; de Souza et al., 2011; Alamy and Bengelloun, 2012; Mendes-da-Silva et al., 2014; Claycombe et al., 2015). However, pups born to LP dams showed no significant effect on body weight at birth. Although we have not estimated the difference in milk production and composition between the HP and LP lactating mothers earlier studies have reported a significant low milk production in the LP group (Moretto et al., 2011). However, we did not observe any difference in lactation time or feeding behavior. A permanent compromise in body and brain weights was noticed in LP group rats from PND3 onwards till the last study time-point and the malnourished rats did not reach the average body weight gain of control rats. Lactational but not gestational protein malnutrition induces bodyweight loss is a novel finding from our study. This loss in body weight in the LP rats can directly be correlated to the increased oxidative stress and excessive breakdown of tissue proteins as reported in PMN conditions (de Belchior et al., 2012; Schiavone et al., 2013; Caballero et al., 2015). The brain weight was significantly low during P5–P17. The brain weight however at birth and P21 was similar in both the control and LP group pups. The similar body and brain weight in both HP and LP pups at birth is considered as fetal adaptation (Gluckman and Hanson, 2004; Alamy and Bengelloun, 2012; Akitake et al., 2015). Similar brain weight of both HP and LP pups at P21 indicates that the brain's high energy content is maintained despite of low energy at the expense of the body.

Maturation of neurological reflexes and other developmental landmarks studied in rats are aspects that characterize postnatal development (Lubics et al., 2005; Barbosa et al., 2015). These tests appear to be the sensitive indicators of the changes induced by environmental adversities like protein malnutrition, infections or other toxicological agents in the intra- and extra- uterine environments (Ten et al., 2003; Belluscio et al., 2014). Decreased bodyweight as well as brain weight during the pre-weaning and permanent compromised postnatal bodyweight in LP group rats in our studies suggest physical slowing and possible deleterious effects of protein malnutrition. Thus, from birth to weaning, the neuro-behavioral evolution of the low and high protein group F1 generation animals was investigated by using a battery of the sensorimotor and neuro-reflex tests. No significant difference was noticed in apparition and performance in surface righting reflex between LP and HP group pups; rather the early appearance of this reflex and better performance in LP group pups can be related to an adaptive necessity of locating and maintaining a suckling station. The neurodevelopmental studies revealed a significant delay in apparition of reflexes in LP pups as compared to the controls. A 2 day delay in apparition of the negative geotaxis reflex was noticed in LP progeny with a 100% attainment of this reflex only by PND 9 with respect to PND 7 in HP controls. Similarly a 24 h delay was recorded in cliff avoidance reflex attainment suggesting delayed neuro-reflex maturation in LP pups. Our results revealed that protein malnutrition induced physical slowing and retarded neuro-behavioral development in the pups characterized by delayed appearance of neurological reflexes (negative geotaxis, cliff avoidance). Several research reports with rat models have already established that maternal protein malnutrition alters morphological, anatomical, and functional characteristics of brain like loss of brain weight, impaired neurogenesis, altered hippocampal formation, neurotransmitter system, and myelination events (Morgane et al., 2002; Steiger et al., 2003; Adebayo et al., 2014; Amaral et al., 2015) that has also been observed in human study by Grantham-McGregor and Baker-Henningham (2005). These impairments could be central to the physical and physiological slowing in LP progeny. Although previous rodent studies have reported delayed physical developmental events like eye opening, incisor eruption, hair growth (Zhang et al., 2010; Belluscio et al., 2014), contradictory to this, no such significant effects were noticed in our study.

Epidemiological investigations have established a correlation between early life nutritional restriction/deprivation and subsequent acquisition of neuropsychiatric illness like depression and/or schizophrenia (Guimaraes et al., 2008; Tomi et al., 2013; Juruena, 2014). Similar observations are also recorded in experimental studies with rats (Xu et al., 2015). As preliminary validation tests of the present study indicated that LP F1 pups irrespective of sex presented significant developmental delay in appearance and maturation of reflex ontogeny. Therefore, we tried to reveal the unique signature effects of the pregestational protein malnutrition on behavioral profile and cognitive abilities of the offspring by assessing the locomoter activity, anxiety-like, and depression relevant behavior, cognitive integrity at 2, 3, and 6 months of postnatal age. Intra-generational PMN resulted in low anxiety, hyperactivity and decreased inhibition-with-time in novelty exploration as manifested in our Open field and EPM results. Increased locomotion and stereotypic behaviors in LP group animals is an indicative of impaired habituation (Almeida et al., 1994; Bobyn et al., 2005). Time in square analysis from open field tracks with significantly more center time is also interpreted as low anxiety and decreased habituation in LP animals (Carola et al., 2002; Ho et al., 2002). Our study reports that intra-generational PMN induces hyperactivity, decreased inhibition-with-time and low anxiety in F1 pups, all these behavioral endpoints have been recognized as outcomes of neuropsychiatric illnesses as has also been noted in rats (Patterson, 2011) and human (Li et al., 2010; Menet and Rosbash, 2011), thereby validating and strengthening our views on the role of maternal protein as a stressor. Our study also revealed poor neuromuscular strength in LP F1 pups irrespective of sex suggesting the poor neuromuscular setup. The encountered cellular and molecular changes in LP rat brains like altered neurotransmitter system, decreased dopaminergic receptors, lower dopamine levels in hippocampus and hypothalamus (Kehoe et al., 2001; Alamy et al., 2005; Mokler et al., 2007; Soares et al., 2013) as well as alterations in hypothalamo- pituitary-adrenal axis (Levay et al., 2010; Del Arco et al., 2011) are reminiscent of a hyperactive like behavior and may constitute neuropsychiatric conditions. Although the impairments observed were consistent throughout the study period, data from animals of 3 months age group showed modulation. These typical behavioral changes at 3 months could be because of sexual maturity associated hormonal changes and correlates well with the findings from other rodent studies of Bronzino et al. (1996, 1999) suggesting altered modulation of dentate gyrus cell excitability due to protein malnutrition during early development but not at 3 month postnatal age. Previous reports in literature with rodent models of either prenatal or postnatal malnutrition have also recorded alterations in behavior and cognitive abilities (Lukoyanov and Andrade, 2000; Françolin-Silva et al., 2006; Alamy and Bengelloun, 2012; Braga et al., 2014). Intra-generational PMN induced significant and permanent impairments on spatial learning and memory in MWM task. A greater difficulty to locate the platform during acquisition trials revealed poor learning in LP group animals. These impairments may well be correlated with previous findings in rats showing PMN induced anatomical, cellular, biochemical and gene expression alterations in hippocampus (Lucassen et al., 2013; Xu et al., 2015). Similarly, the retention trial results suggested memory impairments in LP F1 progeny. It is believed that along with the anatomical and cellular changes, the decreased amounts of CREB^ser133^, an important transcription factor underlying learning and memory in LP brains (Huang et al., 2003) could be the possible reason for LP induced learning and memory impairments. Our results indicated a consistent increase in percent time spent in open arm and number of open arm entries in EPM test at 2, 3, and 6 months of postnatal age in protein malnourished animals. Since low percent open arm time and open arm entries suggest high anxiety, our results are interpreted as indicative of low anxiety, thus validating the low anxiety results from our open field test. PMN induced low anxiety has been very well established in earlier reports as well (Almeida et al., 1996; Alamy et al., 2005; Reyes-Castro et al., 2012). Low protein induced significant impairments in the behavioral and cognitive abilities. However, a general trend was seen with LP F1 males presenting severe effects as compared to LP females. This gender bias in response may have a basis with previous studies with rats reporting that early life nutritional stress perturbs the CSF metabolic profile favoring the female sex (Donner and Lowry, 2013; Tomi et al., 2013), thus supporting the neuro-behavioral sex specific changes as observed in this study.

The present study is the first of its kind to provide a completely relevant model mimicking the IUGR in human females from low socio-economic profile and underdeveloped countries and investigating a wholesome of the effects at physical behavioral and cognitive development levels in F1 progeny. In summary, we conclude that early life protein deprivation results in later life complications at levels of development and behavioral outcome. Protein malnutrition during pre-gestational, gestational, and postnatal life induces deleterious effects with physical and physiological slowing characterized by bodyweight restriction, neurodevelopmental delays in the F1 pups. Lactational, but not gestational protein restriction results in bodyweight loss suggests the adaptive response of the fetus toward the insult, but this definitely occurs at a compromise in the developmental plan. Hyperactivity with low anxiety or fear levels and poor habituation phenotype of the LP F1 progeny corresponds well with the hallmarks of neuropsychiatric diseases like autism and schizophrenia. Impairments in spatial learning and poor retention at 3 and 6 month postnatal age well correlate with the early onset of Alzheimer's suggesting protein malnutrition increases predisposition to such disorders. The general presence of long term behavioral deficits following intra-generational protein deprivation postnatal age suggest that the neuro-behavioral consequences of LP rats are permanent and a resultant of the significant *in-utero* developmental impairments due to poor maternal protein supplies that too during the spurt growth period. Future research efforts are directed toward designing the nutritional rehabilitation strategies and therapeutics for reversal of these PMN induced neuro-behavioral and cognitive deficits.

### Conflict of interest statement

The authors declare that the research was conducted in the absence of any commercial or financial relationships that could be construed as a potential conflict of interest.
